# Effect of Mixed Meal and Leucine Intake on Plasma Amino Acid Concentrations in Young Men

**DOI:** 10.3390/nu10101543

**Published:** 2018-10-18

**Authors:** Naomi Yoshii, Koji Sato, Riki Ogasawara, Yusuke Nishimura, Yasushi Shinohara, Satoshi Fujita

**Affiliations:** 1 Graduate School of Sport and Health Science, Ritsumeikan University, Kusatsu, Shiga 525-8577, Japan; gr0130vi@ed.ritsumei.ac.jp (N.Y.); ysr15159@fc.ritsumei.ac.jp (Y.S.); 2 Graduate School of Human Development and Environment, Kobe University, Kobe, Hyogo 657-8501, Japan; sato712@people.kobe-u.ac.jp; 3 Department of Life Science and Applied Chemistry, Nagoya Institute of Technology, Nagoya 466-8555, Japan; riki.ogasawara@gmail.com; 4 School of Sport, Exercise and Rehabilitation Sciences, University of Birmingham, Edgbaston, West Midlands B15 2TT, UK; nishimuy87@gmail.com

**Keywords:** leucine concentration, protein intake, mixed meal intake

## Abstract

Dietary protein intake is critical for the maintenance of skeletal muscle mass. Plasma amino acid concentrations increase with protein intake and increases in muscle protein synthesis are dependent on leucine concentrations. We aimed to investigate the effect of a mixed meal and free amino acids intake on plasma leucine concentrations. In this randomized crossover study, 10 healthy young men (age 25 ± 1 years, height 1.73 ± 0.02 m, weight 65.8 ± 1.5 kg) underwent tests under different conditions—intake of 2 g of leucine (LEU), intake of a mixed meal (protein 27.5 g, including 2.15 g of leucine, protein: fat: carbohydrate ratio—22:25:53) only (MEAL), intake of 2 g of leucine immediately after a mixed meal (MEAL-LEU) and intake of 2 g of leucine 180 min after a mixed meal (MEAL-LEU180). Blood samples were collected within 420 min (240 min for LEU only) after intake and changes in amino acid concentrations were evaluated. Although the maximum plasma leucine concentration increased to 442 ± 24 µM for LEU, it was lower at 347 ± 16 µM (*p* < 0.05 vs. LEU) for MEAL-LEU, 205 ± 8 µM (*p* < 0.05 vs. LEU) for MEAL. The maximum plasma leucine concentration for MEAL-LEU180 increased to 481 ± 27 µM and compared to LEU there was no significant difference (*p* > 0.1). The observation that rapid elevations in plasma leucine concentrations are suppressed when leucine is ingested at the same time as a meal suggests that the timing of its intake must be considered to maximize the anabolic response.

## 1. Introduction

Dietary protein intake is important to maintain and increase skeletal muscle mass. Skeletal muscle mass undergoes repeated synthesis and breakdown [1]. Specifically, protein ingestion directly stimulates postprandial muscle protein synthesis [2,3]. Ingested protein, after digestion and absorption, appears as amino acids in blood plasma [2,3]. Plasma amino acids are transported into the muscle, which stimulates muscle protein synthesis by increasing the utilization of amino acids in the skeletal muscle cells [4]. Hence, nutritional intake is critical for muscle protein synthesis and studies are now focusing on strategic nutrition intake methods to increase muscle mass effectively.

Increases in protein intake result in an increased muscle protein synthesis (MPS) [5]. Essential amino acids stimulate muscle protein synthesis [6,7] and a large bolus leucine stimulates the anabolic effects of muscle protein by itself. Wilkinson et al. [8] reported that an intake of 3.42 g of leucine increased mechanistic target of rapamycin complex 1 (mTORC1) activation and subsequent muscle protein synthesis rates. Rieu et al. [9] compared the muscle protein synthesis rates between the ingestion of 29.8 ± 1.1 g of casein protein and the same amount of casein protein supplemented with 4.3 ± 0.1 g of leucine. The plasma leucine concentration increased significantly after the intake of casein supplemented with leucine compared to the intake of casein alone and a significantly high MPS was also observed. Moreover, it was found that the maximum plasma leucine concentration after the ingestion of protein sources containing different amounts of leucine was correlated to the muscle protein synthesis rate [10]. These findings highlight the importance of meal-induced maximal plasma leucine concentrations in increasing muscle protein synthesis.

In the context of daily meals, protein is generally consumed in combination with other nutrients (i.e., carbohydrate and fat) as a mixed meal. Food consumed orally enters the stomach where it is retained and undergoes digestion [11,12]. Thereafter, a mixed meal is discharged from the stomach and reaches the small intestine and appears in the blood after absorption through the intestinal wall [13]. With respect to the rate of discharge from the stomach—referred to as gastric emptying food rheology (i.e., liquid vs. solid food) has been shown to influence the gastric emptying, with solid food slower than liquid meal [14]. Moreover, the intake of nutrients ingested in combination also influence gastric emptying. Among macronutrients, lipids, have slower gastric emptying than other nutrients [11,12]. Frost et al. [11] has demonstrated that addition of 30 g oil to pasta meal will delay the gastric emptying as compared to the intake of pasta alone. Another study with mixed meal also indicated a delayed gastric emptying when egg was replaced with fatty liver [12]. These previous studies suggest that the appearance of amino acids in blood plasma from a mixed meal is affected by the intake of nutrients ingested in combination.

Kim et al., in a comparative study on macronutrient mixed meals containing 26 g of protein, reported that regardless of whether it was animal (egg) protein or vegetable (cereal) protein, the maximum plasma leucine concentration did not rise above 200 µM [15]. Moreover, Hudson et al. [16] reported that the maximum plasma leucine concentration was 244 µM (32 ± 1 µg/mL) after the intake of a macronutrient mixed meal containing 30 g of protein. In contrast, previous studies reported that a maximum plasma leucine concentration higher than 400 µM can be obtained with an intake of 20 g of high-quality purified whey protein [10], or an intake of 30 mg/kg body weight of free leucine (for instance, for a body weight of 70 kg, an intake of 2.1 g) [17]. Based on these findings, it is presumed that compared to the intake of protein alone or free amino acids alone, the intake of dietary protein from mixed meals may result in a lower maximum plasma leucine concentration. However, no study till date has investigated the changes in amino acid concentrations after the ingestion of mixed meals in comparison to those after the intake of a similar amount of free amino acids.

Accordingly, we aimed to compare the changes in the plasma amino acid concentrations after the intake of mixed meals, with the changes in the amino acid concentrations after the intake of the same amount of free leucine as that contained in the mixed meal. Additionally, we investigated the absence or presence of the synergy effect in case of the intake of free leucine after the intake of a mixed meal.

## 2. Materials and Methods

### 2.1. Participants 

Ten healthy young men (age 25 ± 1 years, weight 66 ± 2 kg, height 1. 73 ± 0. 02 m) were enrolled. During the study period, including the washout period, when the tests under the four conditions were conducted, participants were instructed to refrain from participating in strenuous exercise, maintain normal levels of daily physical activity and eat normal meals daily. 

The purpose of the study, measurement items and details of the tests were communicated in advance to the participants both verbally and in written form and those who provided written informed consent were considered for study participation. Prior to the performance of the tests, we confirmed that the participants had no chronic health problems using a questionnaire. The study was approved by the Ethics Committee at Ritsumeikan University (approval number BKC-IRB-2015-032) and was conducted in accordance with the Declaration of Helsinki.

### 2.2. Test Protocol

Participants underwent the tests that were performed using a randomized cross-over design. The test comprised four conditions. A gap of at least one week was maintained between two different test conditions. The following four conditions were randomly applied: intake of 2 g of leucine (Bulk Sports, Leucine Power, Inc., Bulk Sports, Sendai, Japan) alone (LEU), mixed meal (protein 27.5 g, including 2.15 g of leucine, protein: fat: carbohydrate [PFC] ratio = 22:25:53) intake alone (MEAL), intake of 2 g of leucine after a mixed meal (MEAL-LEU) and intake of 2 g of leucine 180 min after a mixed meal (MEAL-LEU180).

**Figure 1:**
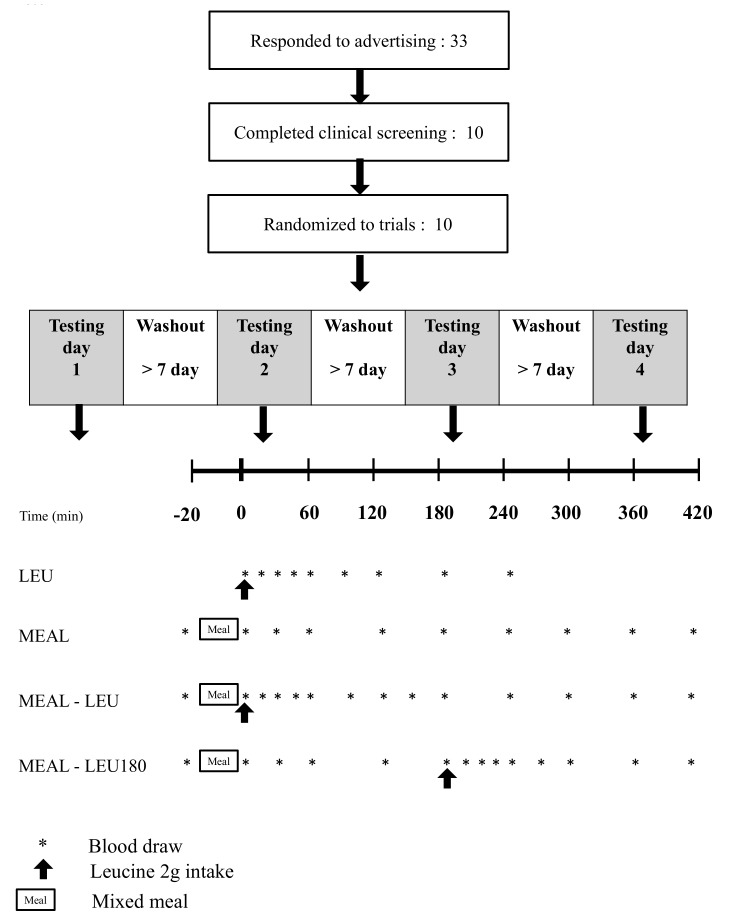
Schematic representation of the study design and timeline. LEU, intake of leucine only; MEAL, intake of a mixed meal only; MEAL-LEU, intake of a mixed meal with leucine intake immediately afterwards; MEAL-LEU180, intake of a test mixed meal followed by leucine intake after 180 min.

The day before the test, the participants ate the same standardized meal (energy 729 kcal, protein 24.1 g, lipid 25.1 g, carbohydrates 97.8 g) for dinner and refrained from eating other foods (except water) until the test was conducted the next morning. At 8 am the following morning, the participants, in a fasted state, reported to the laboratory and remained at rest for 30 min; thereon, venous blood samples were collected through the insertion of an indwelling needle into the cutaneous vein in the forearm. For LEU, the time of blood sample collection in the fasting state was set as time-point 0 min and immediately after sample collection, the participants ingested 2 g of leucine without consuming the test meal. Then, serial blood samples were collected again within 240 min (time point 0–240 min). Under the three conditions involving test meal intake, namely MEAL, MEAL-LEU and MEAL-LEU180, similar to the case of LEU, the participants reported to the laboratory in a fasting state at 8 am and after they remained in a state of rest for 30 min, venous blood samples were collected using the same method as that described above (time-point -20 min). Thereafter, the participants consumed the test meal within 20 min and blood samples were collected (time-point 0 min) immediately after meal intake. Then, blood samples were collected again within 420 min (Figure 1). For MEAL-LUE, the point in time immediately after the intake of 2 g of leucine was set as time-point 0 min. In the case of MEAL-LEU180, 2 g of leucine was consumed (time-point 180 min) after the test meal intake (time-point 0 min). Under all the conditions, the intake of 2 g of leucine was accompanied by the intake of 200 g of water. The participants remained at rest for 240 min or 420 min until blood sample collection was completed.

### 2.3. Test Meal

**Figure 2:**
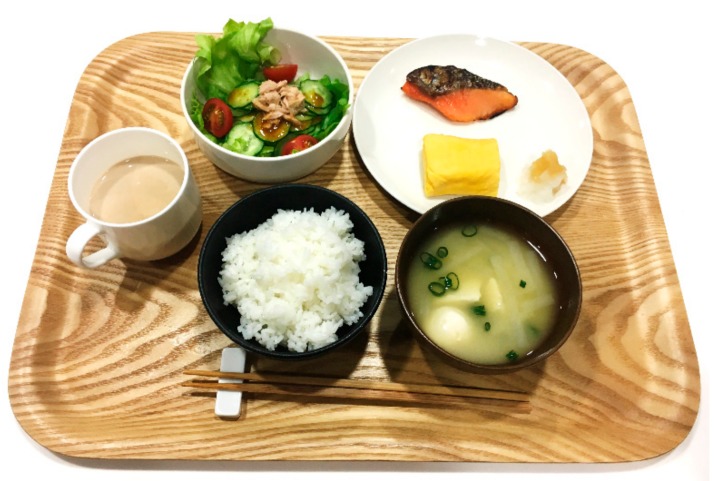
Test mixed meal.

The test meal consumed on the testing day was of Japanese style. The meals were prepared on the day of the test and contained egg, fish and milk for protein, along with other ingredients such as rice and vegetables (Figure 2). The menu comprised grilled salmon, omelet, tuna salad, miso soup, boiled rice and café latte. The meal contained 499 kcal of energy, 27.5 g of protein (including 2.15 g of leucine), 14.1 g of lipids and 65.5 g of carbohydrates and the PFC ratio was 22:25:53. The analysis of the nutritional composition and amount of amino acids such as leucine in the test meal was entrusted to Japan Food Research Laboratories, Tokyo, Japan (Table 1).

### 2.4. Blood Analysis

Blood samples were drawn into vacuum tubes containing EDTA-2Na and after gently tilting to allow mixing, they were immediately cooled in ice water. The cooled sample was then centrifugally separated (4 °C, 3000 rpm, 15 min) and the supernatant plasma was collected. All samples were then stored at −80 °C. 

To determine the plasma amino acid concentrations, centrifugal separation (4 °C, 7000 *g*, 10 min) was performed with the supernatant plasma mixed with 15% sulfosalicylic acid. The supernatant was re-centrifuged (4 °C, 14,000 *g*, 60 min) using an ultrafiltration filter and the lower layers were collected and taken as samples after protein removal [18,19]. Amino acid concentrations were analyzed using a high-speed analyzer (L-8900; Hitachi, Tokyo, Japan). Amino acids were separated using ion exchange chromatography and were detected spectrophotometrically after post-column reaction with ninhydrin. Forty types of amino acids and related molecules were measured, among which the following essential amino acids were used in the analysis: histidine, isoleucine, leucine, lysine, methionine, phenylalanine, threonine, tryptophan and valine.

The insulin concentration in the supernatant plasma samples was measured using an enzyme linked immunosorbent assay (ELISA) kit (Mercodia Ultrasensitive Insulin ELISA kit (Mercodia AB., Uppsala, Sweden) following the guidelines in the reference manual.

Blood glucose levels were measured immediately after blood collection using enzymatic colorimetric endpoint method with Medisafe Fit and Medisafe Fit Chip (TERUMO Inc., Tokyo, Japan).

### 2.5. Statistical Analysis

All values are presented as means ± standard error (SE). A previous study proposed two formulae for the calculation of the area under the curve (AUC): AUC with respect to increase (AUCi) and AUC with respect to ground (AUCg) [20]. In the AUCi, the plasma concentrations in the fasting state and at rest are taken as 0 and the AUC is set as the calculated amount of change in the plasma concentration after the elapsed time. In the AUCg, without setting the plasma concentration in the fasting state and at rest as 0, the AUC includes the area from 0 µM up to the actual concentration. As this study focuses on the amount of change from the state of rest to a point after intake, the AUCi is used. Two separate comparisons were performed for AUCi calculations and statistical analysis. In respect with conditions for total of 2 g leucine intake, LEU and MEAL were compared up to 240 min to reflect the acute meal ingestion. Since 20 min was given for meal ingestion, total length of time for AUCi calculation was 260 min for MEAL (−20 min to 240 min) whereas it was 240 min for LEU (0 min to 240 min). Furthermore, MEAL-LEU and MEAL-LEU180 was compared as conditions for total of 4 g leucine intake. Total length of time for AUCi calculation of MEAL-LEU and MEAL-LEU180 was 440 min (−20 min to 420 min). In the comparison of the four conditions, the presence or absence of a simple main effect was studied using one-way analysis of variance (ANOVA). Afterwards, for cases in which a simple main effect was observed to be significant, the points displaying significant differences were identified using Tukey’s post hoc test. Differences between AUCi values were assessed by a paired t-test. In all the cases, a significance level of 5% was used for two-sided tests. SPSS version 19 (SPSS Inc., Chicago, IL, USA) was used for the analysis.

## 3. Results

### 3.1. Plasma Insulin Concentration

**Figure 3:**
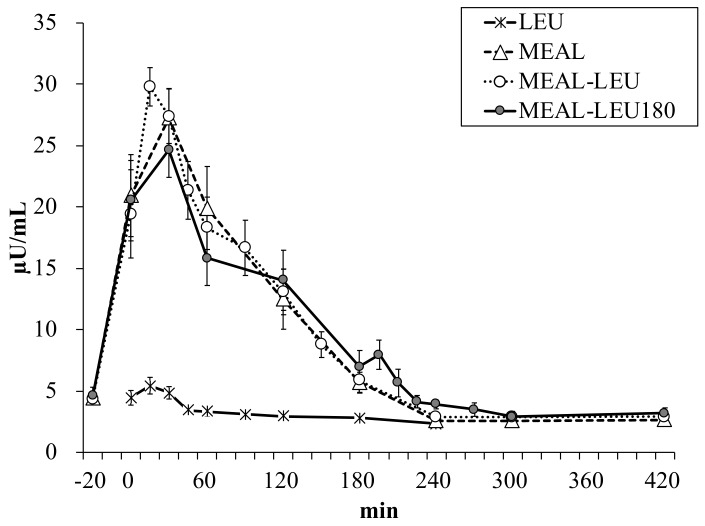
Mean ± SE (*n* = 10) plasma insulin concentration changes over time. LEU, intake of leucine only; MEAL, intake of mixed meal only; MEAL-LEU, intake of mixed meal with leucine intake immediately afterwards; MEAL-LEU180, intake test mixed meal followed by leucine intake after 180 min.

Plasma insulin concentration changes over time is shown in Figure 3. Between the different conditions, there was no significant difference in the plasma insulin concentration in the fasting state (4.4 ± 0.6, 4.4 ± 0.5, 4.4 ± 0.4, 4.6 ± 0.7 µU/mL for LEU, MEAL, MEAL-LEU, MEAL-LEU180, respectively; *p* > 0.1). For LEU, the Cmax value was 6 ± 1 µU/mL and was lower (*p* < 0.05) than that for the other three conditions with mixed meal intake (Table 2). With respect to Tmax, no differences were observed between the conditions. 

### 3.2. Blood Glucose Concentration

**Figure 4:**
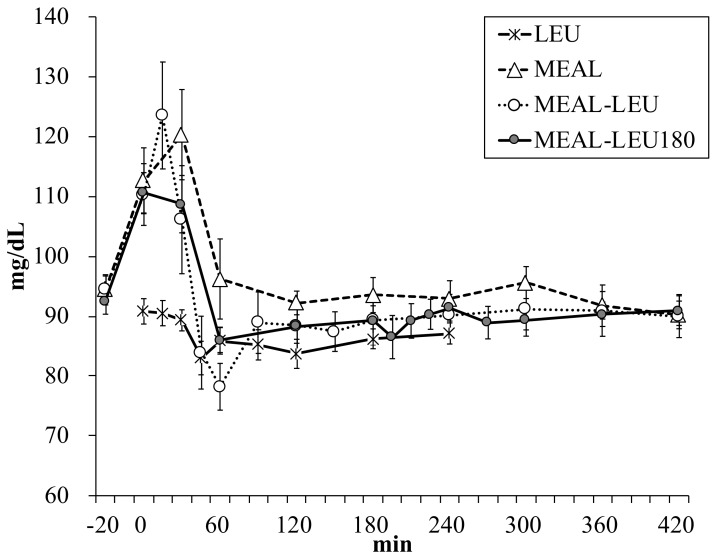
Mean ± SE (*n* = 10) blood glucose concentration changes over time. LEU, intake of leucine only; MEAL, intake of mixed meal only; MEAL-LEU, intake of mixed meal with leucine intake immediately afterwards; MEAL-LEU180, intake of test mixed meal followed by leucine intake after 180 min.

Blood glucose concentration changes over time is shown in Figure 4. Between the different conditions, there were no significant differences in the blood glucose concentrations in the fasting state (91 ± 2, 95 ± 2, 94 ± 3, 93 ± 2 mg/dL for LEU, MEAL, MEAL-LEU, MEAL-LEU180, respectively; *p* > 0.1). The Cmax for LEU was 94 ± 2 mg/dL and was lower (*p* < 0.05) than that for all the other conditions with mixed meal intake (Table 3). For all the conditions, no differences were observed in the Tmax.

### 3.3. Plasma Essential Amino Acid (EAA) Concentration

**Figure 5:**
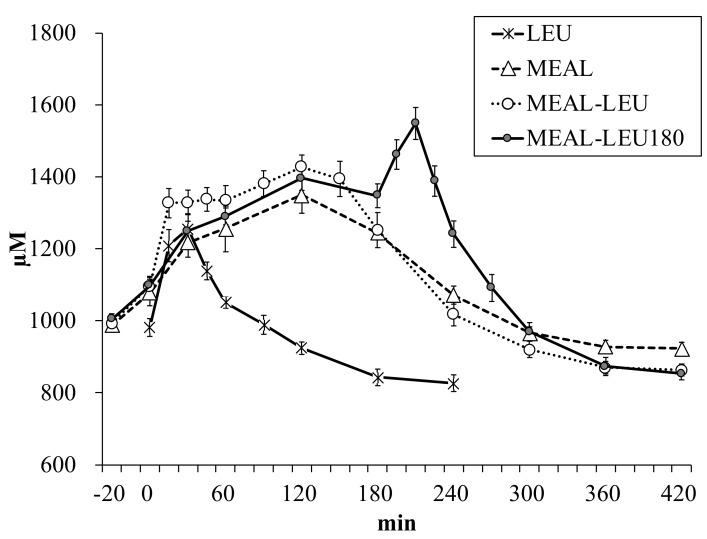
Mean ± SE (*n* = 10) plasma EAA concentration changes over time. LEU, intake of leucine only; MEAL, intake of mixed meal only; MEAL-LEU, intake of mixed meal with leucine intake immediately afterwards; MEAL-LEU180, intake of test mixed meal followed by leucine intake after 180 min.

Plasma EAA concentration changes over time is shown in Figure 5. Between the different conditions, there were no significant differences in the plasma essential amino acid concentrations in the fasting state (982 ± 25, 990 ± 21, 993 ± 15, 1006 ± 14 µM for LEU, MEAL, MEAL-LEU, MEAL-LEU180, respectively; *p* > 0.1). The Cmax was significantly higher (*p* < 0.05) for MEAL-LEU180 than LEU (Table 4). Compared to the cases of MEAL, MEAL-LEU and MEAL-LEU180, the Tmax to reach Cmax was shorter (*p* < 0.05) for LEU. Moreover, there was no significant difference between MEAL and MEAL-LEU. 

### 3.4. Plasma Leucine Concentration

**Figure 6:**
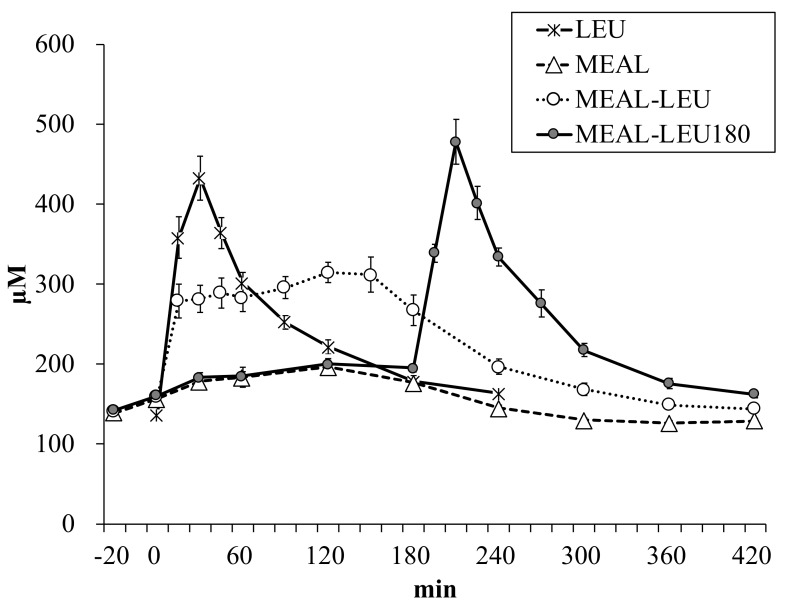
Mean ± SE (*n* = 10) plasma leucine concentration changes over time. LEU, intake of leucine only; MEAL, intake of mixed meal only; MEAL-LEU, intake of mixed meal with leucine intake immediately afterwards; MEAL-LEU180, intake of test mixed meal followed by leucine intake after 180 min.

Plasma leucine concentration changes over time is shown in Figure 6. Between the different conditions, there was no significant difference in the concentration of leucine in the fasting state (137 ± 4, 140 ± 4, 142 ± 4, 143 ± 4 µM for LEU, MEAL, MEAL-LEU, MEAL-LEU180, respectively; *p* > 0.1). The Cmax values for MEAL and MEAL-LEU were lower (*p* < 0.05) than those for LEU (Table 5). There was no significant difference between the concentrations for LEU and MEAL-LEU180. The Tmax to reach Cmax for LEU was shorter (*p* < 0.05) than that for MEAL and MEAL-LEU.

### 3.5. Area under the Curve with Respect to Increase (AUCi) for Plasma Essential Amino Acids and Leucine

In respect with total leucine content of 2 g, AUCi for LEU and MEAL are shown in Table 6. AUCi for essential amino acids. AUCi for essential amino acids was significantly higher for MEAL than LEU (*p* < 0.05). On the contrary, leucine AUCi was significantly higher for LEU as compared to MEAL (*p* < 0.05). 

In respect with total leucine content of 4 g, AUCi for MEAL-LEU and MEAL-LEU180 are shown in Table 7. There was no significant group difference in AUCi for essential amino acids or leucine between MEAL-LEU and MEAL-LEU180. 

## 4. Discussion

In this study, the maximum plasma leucine concentration was higher for free leucine intake alone than mixed meal intake containing the same amount of leucine. We could not observe the presence of a synergy effect with respect to the maximum plasma leucine concentration when free leucine was ingested together with a mixed meal to increase the plasma leucine concentration, suggesting that the intake of a mixed meal and free amino acids together likely delays the digestion and absorption of free amino acids.

To compare LEU and MEAL, the test meal was prepared such that it contained 2 g of leucine to match the free leucine intake of 2 g alone. The amount of protein in the test meal was 27.5 g because of this consideration. Previous studies have reported rapid increases in plasma leucine concentrations with free amino acids or whey intake [2,10,17]. However, in experiments with the intake of mixed meals that are very similar to normal daily meals [15,16,21], a rapid increase in plasma amino acid concentrations were not observed. This is in agreement with the findings of our study. Compared to the intake of free leucine alone, for the intake of mixed meals containing the same amount of leucine, the maximum plasma leucine concentration was significantly lower (Table 5). The Cmax after a mixed meal intake was about 47 ± 2% of the value obtained after free leucine intake alone.

Tmax, the time taken to reach the maximum plasma leucine concentration, showed a significant delay for MEAL compared to LEU (Table 5). This could be attributed to the fact that, in mixed meal intake, the food is retained in the stomach before gastric emptying. The rate of gastric emptying is known to be affected by food rheology (i.e., liquid or solid) [14] and nutrients ingested at the same time (lipids in particular) [11,12]. According to Frost et al. [11], gastric emptying was not delayed, when fiber (1.7 g psyllium) was added to pasta with tomato sauce. On the contrary, when 30 g sunflower oil was added to pasta with tomato sauce with or without fiber (1.7 g psyllium), then the gastric emptying was delayed. Therefore, fat ingestion may be a more influential factor for gastric emptying than fiber ingestion. Since normal daily meals (mixed meals) always contain both protein and other nutrients, gastric emptying is delayed, leading to increased time to appear in the plasma after digestion and absorption in the small intestine; this in turn likely causes the delay in the plasma leucine concentration increase.

The AUCi of the plasma leucine concentration was significantly lower for MEAL than LEU (Table 6), although total length for calculating AUCi was 20 min longer in MEAL compared to that of LEU. The AUCi assesses the amounts of amino acids that appear in circulation after digestion and absorption. Egg, fish, soy products and milk, considered high-quality proteins according to the digestible indispensable amino acid score (DIAAS) [22], were used for the protein content in the test mixed meal used in this study. These dietary proteins were selected based on a typical Japanese breakfast. Even if proteins with a high DIAAS are ingested, the entire ingested amount may not ultimately reach circulation. The digestion and absorption of protein involves the retention of amino acids in the small intestine and liver (first-pass splanchnic extraction) facilitating absorption [2,13,23,24,25]. It should be noted that branched-chain amino acids, including leucine, from beef protein have been shown to escape hepatic uptake and/or metabolism after intestinal absorption [26]. Regardless, Capaldo et al. [13] reported that in the case of protein intake from meals containing various nutrients, the amount of amino acids appearing in the blood stream after digestion and absorption is only 30% of the intake amount. The undigested portion of the ingested protein remains in the intestine even five hours after intake and more than half of the intake amount does not appear in the blood stream because of first-pass splanchnic extraction, the rate of which is reported to about 73.0 ± 1.4% of the intake amount for casein and about 66.1 ± 1.2% for casein hydrolysate. Since the first-pass splanchnic extraction rate for casein hydrolysate is significantly lower than that for casein, the appearance rate in the plasma is higher for casein hydrolysate [23]. Moreover, Penning et al. [2] reported that the amount of amino acids appearing in the blood stream after the digestion and absorption of ingested whey protein is only about 60% of the intake amount; the remaining about 40% does not appear in the blood stream even four hours after ingestion due to first-pass splanchnic extraction. In tests with the intake of 40 g of a free amino acid mixture, the first-pass splanchnic extraction rate was 29 ± 5% in young adults and 47 ± 3% in elderly people, confirming that first-pass splanchnic extraction also occurs in the case of free amino acid intake, preventing the full amino acid amount from appearing in the blood stream [24]. However, while age is a factor in young adults and elderly people, the first-pass splanchnic extraction rate may be low in the case of highly purified amino acid intake compared to dietary protein intake. Accordingly, the AUCi was lower for the mixed meal intakes than free leucine intake alone in our study, not only due to delays in gastric emptying owing of the lipids [11,12] ingested at the same time but also likely due to first-pass splanchnic extraction during digestion and absorption. 

The maximum plasma leucine concentration for conditions involving the intake of free leucine in addition to mixed meals was found to be significantly higher for MEAL-LEU180 than MEAL-LEU (Table 5). Since food is retained in the stomach and gastric emptying is delayed after mixed meal intake [11,12], free leucine might have remained in the stomach along with the other foods ingested, preventing it from appearing in the plasma through rapid absorption; MEAL-LEU may have led to the lower Cmax values. However, significant gastric emptying occurs by 2 h after mixed meal ingestion [12,27,28]. MEAL-LEU180 is likely that the free leucine ingested after 180 min was rapidly emptied from the stomach to the small intestine, causing it to appear faster in the plasma. Moreover, there was no significant difference in the Cmax values between MEAL-LEU180 and LEU. These results show that rapid absorption, as in the case of free leucine intake in a fasting state, also occurred when free leucine was ingested 180 min after a mixed meal, causing its rapid appearance in plasma.

The present study has some limitations. The increase in the plasma amino acid concentration is not just a result that digestion and absorption of orally ingested mixed meal. It is a net balance of amino acids taken up and released by the various organs. Since stable isotopes were not used in the study, the accurate amino acid absorption kinetics or amino acid metabolism status from free leucine or mixed-meal could not be assessed [2,23]. However, the purpose of this study was to compare their state of appearance in the plasma by ensuring the free amino acid supplement intake amount was the same as the amount of amino acids contained in the test mixed meal. Accordingly, without the use of whey or other EAAs contained in amino acid mixtures, a comparative investigation was conducted by matching the free leucine intake amount with the leucine content in the test mixed meal.

## 5. Conclusions

Based on the aforementioned discussions, the intake of free leucine alone markedly increased the plasma leucine concentration. However, the increase in leucine concentration after the intake of a mixed meal containing the same amount of leucine was significantly less than that of free leucine intake alone. Moreover, when free leucine was ingested after a mixed meal with the purpose of increasing the plasma leucine concentration, the maximum plasma concentration was attenuated when it was ingested immediately after the mixed meal, despite the fact that the total leucine content was doubled. These results suggest that when free amino acids ingested with the purpose of increasing plasma amino acid concentrations, the timing in relation to the mixed meal intake needs to be considered.

## Figures and Tables

**Table 1:** Essential amino acid composition of the mixed meals.

	(g)
Histidine	0.91
Isoleucine	1.23
Leucine	2.15
Lysine	1.93
Methionine	0.67
Phenylalanine	1.25
Threonine	1.17
Tryptophan	0.36
Valine	1.50

**Table 2:** Plasma insulin concentration of the pharmacokinetics parameters.

	LEU	MEAL	MEAL-LEU	MEAL-LEU180
Cmax (µU/mL)	6 ±1 ^a^	31 ± 2 ^b^	32 ± 1 ^b^	27 ± 6 ^b^
Tmax (min)	17 ± 4	24 ± 6	18 ± 3	21 ± 17

All data are expressed as means ± SE (*n* = 10). Means that do not share a letter are significantly different (*p* < 0.05). Cmax, maximum concentration; Tmax, time to reach the maximum concentration; LEU, intake of leucine only; MEAL, intake of mixed meal only; MEAL-LEU, intake of mixed meal with leucine intake immediately afterwards; MEAL-LEU180, intake of test mixed meal followed by leucine intake after 180 min.

**Table 3:** Blood glucose concentration of the pharmacokinetics parameters.

	LEU	MEAL	MEAL-LEU	MEAL-LEU180
Cmax (mg/dL)	94 ± 2 ^a^	133 ± 5 ^b^	131 ± 6 ^b^	118 ± 3 ^b^
Tmax (min)	11 ± 4	18 ± 5	11 ± 2	9 ± 5

All data are expressed as means ± SE (*n* = 10). Means that do not share a letter are significantly different (*p* < 0.05). Cmax, maximum concentration; Tmax, time to reach maximum concentration; LEU, intake of leucine only; MEAL, intake of mixed meal only; MEAL-LEU, intake of mixed meal with leucine intake immediately afterwards; MEAL-LEU180, intake of test mixed meal followed by leucine intake after 180 min.

**Table 4:** Plasma essential amino acid concentration of the pharmacokinetics parameters.

	LEU	MEAL	MEAL-LEU	MEAL-LEU180
Cmax (µM)	1273 ± 40 ^a^	1380 ± 39 ^a,b^	1479 ± 35 ^b,c^	1570 ± 39 ^c^
Tmax (min)	27 ± 4 ^a^	132 ± 12 ^b^	108 ± 15 ^b^	220 ± 9 ^c^

All data are expressed as means ± SE (*n* = 10). Means that do not share a letter are significantly different (*p* < 0.05). Cmax, maximum concentration; Tmax, time to reach maximum concentration; EAA, essential amino acid; LEU, intake of leucine only; MEAL, intake of mixed meal only; MEAL-LEU, intake of mixed meal with leucine intake immediately afterwards; MEAL-LEU180, intake of test mixed meal followed by leucine intake after 180 min.

**Table 5:** Plasma leucine concentration of the pharmacokinetics parameters.

	LEU	MEAL	MEAL-LEU	MEAL-LEU180
Cmax (µM)	442 ± 24 ^a^	205 ± 8 ^b^	347 ± 16 ^c^	481 ± 27 ^a^
Tmax (min)	33 ± 3 ^a^	117 ± 14 ^b^	96 ± 17 ^b^	229 ± 2 ^c^

All data are expressed as means ± SE (*n* = 10). Means that do not share a letter are significantly different (*p* < 0.05). Cmax, maximum concentration; Tmax, time of maximum concentration; LEU, intake of leucine only; MEAL, intake of mixed meal only; MEAL-LEU, intake of mixed meal with leucine intake immediately afterwards; MEAL-LEU180, intake of test mixed meal followed by leucine intake after 180 min.

**Table 6:** Area under the curve with respect to increases for essential amino acids and leucine for LEU and MEAL.

	LEU	MEAL
Essential amino acids (µM·min)	−3859 ± 2481	58,848 ± 3050 *
Leucine (µM·min)	25,561 ± 1112	8154 ± 840 *

All data are expressed as means ± SE (*n* = 10). * Indicate values significantly different from LEU (*p* < 0.05). Total length of time for AUCi calculation for LEU was 240 min (−20 to 240 min), whereas it was 260 for MEAL (−20 min to 240 min). LEU, intake of leucine only; MEAL, intake of mixed meal only; MEAL-LEU, intake of mixed meal with leucine intake immediately afterwards; MEAL-LEU180, intake of test mixed meal followed by leucine intake after 180 min.

**Table 7:** Area under the curve with respect to increases (total Leucine intake of 4 g groups).

	MEAL-LEU	MEAL-LEU180
Essential amino acid (µM·min)	59,318 ± 5812	73,167 ± 6848
Leucine (µM·min)	35,847 ± 2982	35,167 ± 1735

All data are expressed as means ± SE (*n* = 10). Total length of time for AUCi calculation for MEAL-LEU and MEAL-LEU180 was 440 min (−20 to 420 min). MEAL-LEU, intake of mixed meal with leucine intake immediately afterwards; MEAL-LEU180, intake of test mixed meal followed by leucine intake after 180 min.

## Abbreviations

The following abbreviations are used in this manuscript:

AA	Amino acid
AUCi	Area under the curve with respect to increase
Cmax	Maximum concentration
EAA	Essential amino acid
MPS	Muscle protein synthesis
Tmax	Time of maximum concentration
